# Use of the lambda Red recombinase system to produce recombinant prophages carrying antibiotic resistance genes

**DOI:** 10.1186/1471-2199-7-31

**Published:** 2006-09-19

**Authors:** Ruth Serra-Moreno, Sandra Acosta, Jean Pierre Hernalsteens, Juan Jofre, Maite Muniesa

**Affiliations:** 1 Department of Microbiology. Faculty of Biology. University of Barcelona. Diagonal 645. E-08028 Barcelona. Spain; 2 Viral Genetics Laboratory, Faculty of Sciences, Vrije Universiteit Brussel, Pleinlaan 2, B-1050 Brussel, Belgium

## Abstract

**Background:**

The Red recombinase system of bacteriophage lambda has been used to inactivate chromosomal genes in *E. coli *K-12 through homologous recombination using linear PCR products. The aim of this study was to induce mutations in the genome of some temperate Shiga toxin encoding bacteriophages. When phage genes are in the prophage state, they behave like chromosomal genes. This enables marker genes, such as antibiotic resistance genes, to be incorporated into the *stx *gene. Once the phages' lytic cycle is activated, recombinant Shiga toxin converting phages are produced. These phages can transfer the marker genes to the bacteria that they infect and convert. As the Red system's effectiveness decreased when used for our purposes, we had to introduce significant variations to the original method. These modifications included: confirming the stability of the target *stx *gene increasing the number of cells to be transformed and using a three-step PCR method to produce the amplimer containing the antibiotic resistance gene.

**Results:**

Seven phages carrying two different antibiotic resistance genes were derived from phages that are directly involved in the pathogenesis of Shiga toxin-producing strains, using this modified protocol.

**Conclusion:**

This approach facilitates exploration of the transduction processes and is a valuable tool for studying phage-mediated horizontal gene transfer.

## Background

The analysis of microbial sequences has revealed that a substantial fraction of the genome of some bacteria corresponds to prophage DNA [1,2]. Such prophage DNA is inserted into the bacterial chromosome after infection by free phage particles. Bacteiophages have recently regained part of their former importance, but now with a medical and ecological focus.

Phage λ DNA integrates into host DNA at one preferential site in *Escherichia coli*. This integration occurs through homologous recombination, mediated by the action of site-specific recombinases [3]. Bacteriophage can be released from cells containing an intact prophage through a process called induction. In this process, the prophage genes required for lytic growth are turned on, and progeny virions are produced and released from the cell. Cells carrying a prophage are called 'lysogens' because of their potential to be induced and to lyse [2,4]. Most phage genes, including those required for lytic growth and virion production, are turned off in integrated prophages. However, prophages express regulatory proteins involved in the maintenance of the lysogenic state and 'lysogenic conversion' genes which alter the properties of the host bacterium. The products of these genes may have strong effects on the host bacterium, which can have its phenotype modified by expression of genes encoded by the prophage. These changes range from protection against further phage infection to increasing the virulence of a pathogenic host.

The presence or absence of prophages can account for much of the variation among individuals within a bacterial species. In addition, phages are likely to be important vehicles for horizontal transfer of genetic information between bacteria [2,5-7]. Clearly, to understand fully the role of phage-mediated gene transfer in the evolution of pathogenic bacteria, it is essential to study and understand the mechanisms of phage-mediated gene transfer itself.

To evaluate the mechanisms of lysogeny and phage-mediated horizontal gene transfer, recombinant phages carrying a marker gene, such as GFP (green fluorescent protein) [8] or antibiotic resistance cassettes [9], have been proposed as a useful tool. Such phages would convert the host bacteria after infection and integration, making the lysogens easy to detect in further studies.

A number of general allele replacement methods can be used to generate recombinant *stx*-phages. Some of these require creating a gene disruption in a suitable suicide plasmid before recombining it into the chromosome. The key to this procedure involves the use of vectors that cannot replicate under conditions used for selection of the cointegrates. Examples of such vectors for *E. coli *include ColE1-derived plasmids, which do not replicate in *polA *mutants [10], a temperature-sensitive pSC101 replicon [11], and a phagemid-based vector [12]. The application of *pir*-dependent vectors, as used to generate φ3538, another stx::*cat *recombinant phage [9], and *repA*-dependent broad-host-range plasmids for use in Gram-positive bacteria [13], have also been described. The suicide plasmid carrying the cloned mutant sequences is transferred in the host cells. At the non-permissive conditions for plasmid replication, cells maintain antibiotic resistance only if the plasmid integrates into the chromosome by homologous recombination between the cloned fragment and the bacterial chromosome. Excision of the integrated plasmid takes place by a second homologous recombination, integrating the desired mutation into the bacterial genome, and could be sometimes selected for antibiotic sensitivity or resistance to the presence of sucrose in the agar [14]. Allison *et al*. [15] produced recombinant phages by the creation of a recombinant plasmid harbouring the mutagenic template. Homologous recombination takes place between the replicating plasmid bearing the genetic construct and either the integrated prophage, the new replicated phage genome copy or the incoming viral genome. In the last two situations, DNA would have to be in the circular intermediate form. This method avoids the use of a suicide vector and recombinant phages were identified at a very high frequency. Similar approaches were also applied by other authors [8]. The mini-transposon Tn *10d-bla*, was used to produce beta-lactamase fusions to phage-encoded, exported proteins [16].

Some methods have been also developed to introduce linear DNA in Gram-positive or Gram-negative bacteria [17-19]. In parallel, Datsenko and Wanner [20] proposed a method for inactivating chromosomal genes in *E. coli *K-12 in one step, by recombining a PCR product using the lambda Red system (γ, β, *exo*). This enhances the recombination of linear DNA in the *E. coli *chromosome, as it promotes efficient double-strand break repair/recombination. This method is analogous to that used for many years in yeast [20]. It is a useful method for constructing mutants, avoiding the use of suicide plasmids or the previous generation of plasmids carrying the constructs. Several authors have successfully applied this method for different purposes [21,22].

Some bacterial virulence factors, such as toxins or antibiotic resistance genes, are frequently encoded by bacteriophages. For example, factors encoded by phages have been described in some of the emerging or reemerging pathogens. These include: pyrogenic exotoxin A production in group A streptococci [23]; cholera toxin in *Vibrio cholerae *[24]; betalactamase genes [25]; and the production of Shiga toxin, the subject of this study and one of the most important virulence factors in Shiga toxin-producing strains of *E. coli *(STEC). The toxin genes are encoded by the genome of temperate lambdoid bacteriophages or *stx*-phages [26-28]. The use of the Red recombinase methodology can be applied to introduce marker genes in the prophage genomes, within the non-essential toxin genes, as prophage genes behave like chromosomal genes. Here we present an approach based on the use of the Red recombinase system to construct Shiga toxin-producing bacteriophages carrying antibiotic resistance genes.

## Results and Discussion

### Modification of the Red recombinase methods

Our first attempts at using the protocol described by Datsenko and Wanner [20] to construct recombinant prophages were unsuccessful. After several trials, we decided to modify the protocol. The modifications allowed the use of this methodology with the special characteristics of our strains, which carry inducible Shiga toxin-converting prophages.

The most obvious drawback was the spontaneous activation of the phage's lytic cycle during the process. This affirmation is supported by the isolation of phage DNA from the supernatants of the bacterial cultures without previous induction (data not shown). This increased prophage excision and/or phage release, which significantly reduced the efficacy of recombinant clone formation. Two possible causes of this failure were considered. Possibly, excision of the prophage DNA from bacterial DNA occurred without phage particle formation. In this case, the target gene (*stx*) where the amplimer must recombine would be lost, thus hindering correct recombination. Alternatively, after excision, phage particles were formed and released from the cells by lysis. In this case, a significant proportion of bacterial cells would be lysed, reducing the number of cells susceptible to transformation. Both of these scenarios would lead to a reduction in the efficacy of obtaining recombinant clones. The first cause could not be checked, but the second one was experimentally confirmed by the moderate reduction of the OD_600 _observed in the cultures of the lysogens during the process, compared with the OD_600 _of *E. coli *C600 or DH5α cultures (non-lysogens) used as a control.

The spontaneous induction of the lytic cycle could be due to several causes that would activate the SOS response. For example, the double application of the protocol to prepare electrocompetent cells (firstly to transform plasmid pKD46 and secondly to transform the PCR amplimer) which may activate the stress response in the bacterial cell. In any case, we assayed several modifications of the original protocol to optimize our application. These modifications of each step and the results obtained are described in this section.

#### Confirmation of the presence of the *stx *genes after pKD46 vector transformation

We examined the efficacy of the transformation of the helper plasmid pKD46 in the first step of the method, to evaluate the efficacy of our transformation protocol. Vector pBC-SK was used as a control. Vector pKD46 presented a lower transformation efficacy than pBC-SK. The number of transformed colonies was on average 6–10 fold higher with pBC-SK. These results could be due to the fact that pKD46 carries a temperature-sensitive origin of replication and only replicates at 30°C. Therefore the growth rate of cells transformed with pKD46 is lower than that of cells transformed with pBC-SK and grown at 37°C.

In addition to these expected results, the introduction of the pKD46 vector produced the loss of the *stx*_*2 *_gene in some of the lysogens. This was observed in a high proportion of the analyzed colonies (Table 1). The percentage of gene loss in such lysogens was significantly higher (*t*-Student, *p* < 0.05) than the percentage of *stx*_*2 *_gene loss in transformed cells after transformation with vector pBC-SK, used as control (Table 1). Only lysogens C600(933W) and C600(A9) did not show any gene loss which could suggest a higher stability of lysogens obtained with strain C600.

Since the *stx*_*2 *_gene was required to continue with the protocol, the presence of the vector and the *stx*_*2 *_gene in the selected clones was confirmed by a double hybridization with the respective *stx *and Red recombinase probes. Only clones in which the pKD46 and the *stx*_*2 *_gene were observed could be used in the next steps to transform the amplimer containing the antibiotic gene.

Other phage sequences, such as the *rho *independent terminator, the *cI *gene or the *Q *gene, which are present in the studied phages, were also absent in colonies lacking the *stx*_*2 *_gene (data not shown). This suggests that excision of the whole prophage DNA from the host cell occurred. However, since this was not the aim of this work, further investigations were not undertaken.

There is no explanation for the loss of the *stx *gene in a high percentage of the cells transformed with pKD46. Conditions were the same for all lysogens and some of them did not lose the *stx*. A possible hypothesis is that the presence of the Red recombinase system could increase the excision of certain prophages from the bacterial genome of some lysogens, without virus formation and without subsequent cell lysis.

#### Construction of the amplimer

One of the most important modifications to the protocol involved the construction of the PCR amplimer. In the first set of experiments, we used two primer sets to amplify the resistance marker containing each 36 bp and 40 bp extension sequences (short homologous arms) with homology to the *stx *genes (Table 2). These shared sequences were expected to be effective with the Red recombinase system. The primers were designed to obtain a Tc amplimer and a Cm amplimer that could be inserted into the recombinant phages. However, no antibiotic-resistant transformants of our lysogens were obtained using these primers. Therefore they were excluded from the experimental procedure.

The presence of Tc-resistant transformants was only observed on a few occasions when using the above described primers. However, the Tc cassette was not present inside the *stx*_*2 *_gene but somewhere else in the bacterial chromosome, despite the fact that all the plasmids used as templates of the antibiotic cassettes had conditional replicons to avoid erroneous integrations as suggested in the original protocol. Digestion of the amplimers with *DpnI *was to reduce this problem did not improve the results. The use of short homology sequences (36–40 bp) for the recombination could possibly be the cause of the wrong recombination events or the lack of recombinations. For this reason, the length of the homology regions where the recombinase can act, was increased. For this purpose a new strategy, based on the 3S-PCR protocol [29] was used to increase the length of the homology region shared by the amplimer and the *stx*_*2 *_gene.

The unusual recombination events are difficult to explain. Conceivably, the presence of the *stx *lambdoid prophages, some of which could include different recombinase genes, might somehow enhance non-homologous recombination. This would lead to the insertion of the antibiotic resistance cassette in unexpected sites outside of the target gene. In fact, the sequencing of several *E. coli *genomes has revealed the widespread occurrence of multiple integrases of phage origin [30] with similar sequence homology. These can help to explain our observations, which have also been described by other authors [31].

**Figure 1:**
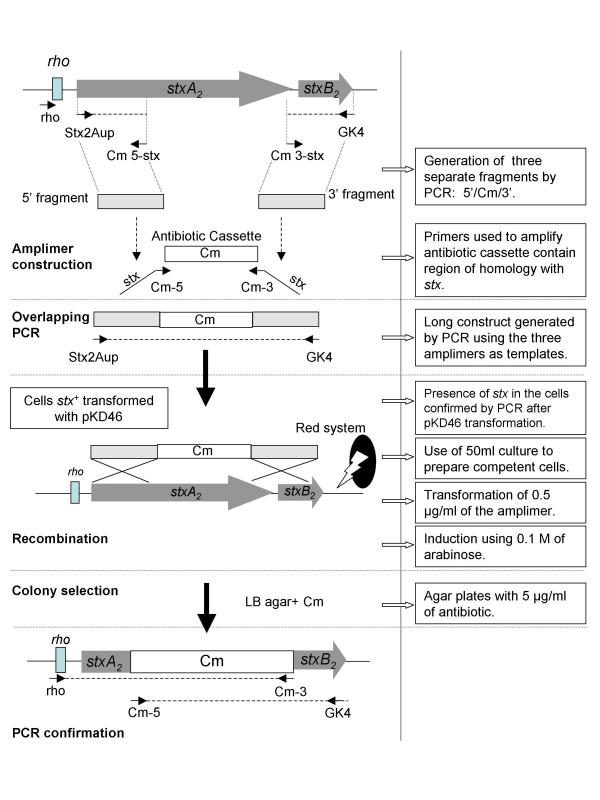
Scheme of the protocol used to construct the amplimers containing the *stx *gene substituted by the antibiotic resistance gene. The figure shows the protocol used for the *cat *gene as an example.

To increase the length of the *stx*_*2 *_homologous region upstream and downstream of the antibiotic resistance cassette, new amplimers were created by using overlapping regions between three different fragments: the 3' fragment, containing the *stx*_*2 *_homology; the antibiotic resistance cassette; and the 5' cassette, which also contains the *stx*_*2 *_homology (Fig 1). Some amplimers, such as the one containing *tet*, were directly obtained using the three fragments simultaneously as templates in the same PCR reaction. For *cat*, the 3'-fragment joined to the antibiotic cassette was amplified in a first PCR reaction and the antibiotic cassette joined to the 5'-fragment in a second one. Then both fragments were used together to obtain the complete amplimer in a third PCR reaction.

The use of longer homologous regions (280 bp at each side) in the amplimer carrying the antibiotic cassettes definitely solved the problem of false recombinants (Table 2). In the above mentioned application (29), the rational for the use of longer regions of homology was to increase the probability of the double event of recombination that lead to the desired allelic exchange in non-*E. coli *K-12 bacteria. This was explained by the authors since the lambda-red functions became less efficient in blocking DNA degradation in bacteria that are distantly related to *E. coli*. Therefore, longer regions of homology increased the efficiency. Although our application was performed using an *E. coli *K-12 derivative, strain DH5α, the same strategy of long arms seems to be suitable for our purposes.

The amount of the amplimer to be transformed was also evaluated and finally established at 0.5 μg of amplified DNA (Table 2). From 10–100 ng of amplified DNA were established in the original protocol [20]. However, in our hands and using an amplimer longer than the one described in the original protocol, higher concentrations were necessary.

#### Transformation of the amplimer

Initially, we used 5 ml SOB cultures with ampicillin and 10 mM of L-arabinose as described by Datsenko and Wanner [20]. However, no recombinant clones were obtained. In successive attempts, lysogenic cells (pKD46^+^, *stx*_*2*_^+^) in culture volumes of 10, 25 and finally 50 ml were used to prepare electrocompetent cells (Table 2). Electrocompetent cells prepared from 50 ml of culture were finally used to obtain recombinant clones. This problem did not apply in the approach described by Datsenko and Wanner [20], but in our approach it was necessary to obtain few recombinant colonies. This confirmed our hypothesis that in our cultures the initial number of cells was probably reduced by the activation of phage lysis during the protocol for preparation of electrocompetent cells or by the presence of antibiotic in the culture media. Therefore the minimal numbers of cells in the initial culture necessary to obtain a single recombinant colony was of 2.5 × 10^10 ^CFU/ml.

Different concentrations of L-arabinose were also tested. In optimal conditions, no significant differences were observed in our experiments when using different amounts of arabinose. We assayed 1 mM (indicated in the original protocol), 10 mM and 0.1 M of L-arabinose. Recombinant clones were obtained in all cases. However, the highest number of clones was obtained with 0.1 M of arabinose (Table 2). These results indicate that all of the concentrations tested were inside the range needed to generate the expression of the γβ*exo *gene system in a proportion of the transformed cells. This is required in a system that is dependent on arabinose concentrations and that has been described as "all-or-none induction of P_BAD_" [32].

After transformation of the amplimer, the cells were recovered in 1 ml of SOC medium and incubated for 1 to 3 hours at 37°C (as suggested in the original method) or 30°C before plating. Three hours of incubation were required to obtain recombinants. Although pKD46 is supposed to be a temperature sensitive vector and cannot replicate above 30°C, the proteins encoded by the vector still can be active inside the competent cells enhancing recombination. There were no great differences in the number of clones obtained from incubation at different temperatures (Table 2) although more colonies were obtained at 37°C. In fact, incubation at 37°C increased the growth rate, producing more cells.

#### Plating media

LB agar plates containing Tc and Cm were used as selective media. Datsenko and Wanner [20] proposed the use of 25 μg/ml for Cm and Km. Initially, we tested concentrations of 20 μg/ml Tc or 20 μg/ml Cm for the recovery of recombinants. However, no recombinants were observed on Cm or Tc plates (Table 2). Positive results were obtained when using lower antibiotic concentrations in the selective media. Therefore, LB plates containing 5 μg/ml Tc or 5 μg/ml Cm were finally used for the recovery of recombinants. Once plated, 24 hours of incubation were enough to visualize colonies. The colonies were then transferred to new LB agar plates with higher antibiotic concentrations (Tc: 20 μg/ml and Cm: 20 μg/ml respectively). Thus lysogens carrying recombinant phages did not grow immediately in the expected antibiotic concentrations, possibly be because bacterial cells need to recover after the process, and were damaged when directly submitted to high antibiotic concentrations.

Loss of the vector was achieved after successive subcultures and incubation at 43°C without ampicillin selection, as previously described [20]. The loss of the plasmid was confirmed by observing the absence of the PCR amplimer, using the primers described in Table 1 for vector pKD46.

Table 2 summarizes all the variations assayed in our protocol; the number of recombinant clones obtained with two of the bacteriophages using the different modifications; and the final conditions that were established.

### Recombinant phages

The *tet *and *cat *genes were introduced in the *stx*_*2 *_gene of prophages: ØA9, ØA312, ØA534, ØA549, ØA557, ØVTB55 and 933W. The genes were placed at position 251 bp of the *stx*_*2*_-*A *subunit and 264 bp upstream of the end of the *stx*_*2*_-*B *subunit. The identification and characterisation of the respective gene in each recombinant prophage was achieved by PCR and sequencing.

The induction of *stx*_*2*_-phages from the six recombinant lysogens had slightly different kinetics than the original lysogens. Nevertheless, all recombinant lysogens conserved their lytic capacity after incorporating the antibiotic resistance gene. As previously described [33,34], plaques produced by *stx*-phage are usually poorly visible or turbid on the top agar layer. Therefore, confirmation of the presence of infective recombinant phages was achieved by plaque blot hybridization with specific *cat *or *tet *probes (data not shown).

The antibiotic resistance cassette appears to remain stable within the phage genome, as observed after four steps of subculture without antibiotic selection. However the long-term stability of the marker gene in the phage genome without antibiotic selection remains to be elucidated.

### Recombinant transductants

**Figure 2:**
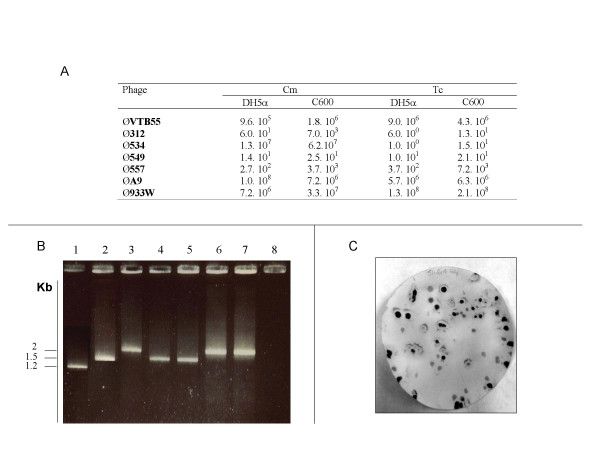
A) Number of transductants (CFU/ml) obtained in *E. coli *DH5α and *E. coli *C600 with each recombinant phage carrying *ca*t or *tet *antibiotic resistance genes. B) PCR products of each recombinant colony carrying a stx-prophage with the respective antibiotic resistance cassette. 1: *stx *gene control, 2: *cat *control, 3: *tet *control, 4–5: *E. coli *DH5α and *E. coli *C600 lysogens with *stx*-phages::*cat*. 6–7: *E. coli *DH5α and *E. coli *C600 lysogens with *stx*-phages::*tet*-. 8 negative PCR control; C) As an example, colony blot of DH5α (Ø557::*tet*) hybridized with the specific probe for the *tet *gene.

To evaluate the capacity of the recombinant phages to infect and convert new *E. coli *strains, suspensions of the recombinant phages were prepared. Transduction of *E. coli *DH5α and *E. coli *C600 was performed. All phages produced new transductants, which conferred resistance to the appropriate antibiotic on DH5α or C600. Presence of the recombinant prophages in the host strain was confirmed by PCR and plaque hybridization analysis (Figure 2).

Some other authors have produced recombinant *stx*-phages [9,15,16] for different purposes. Schmidt *et al*. [9] used phage φ3538 to infect and lysogenize enteric *Escherichia coli *strains and to develop infectious progeny from such lysogenized strains. Allison *et al*., [15] used recombinant phages to show the first reported observation of the simultaneous infection of a single host with two genetically identical Stx phages. Acheson *et al*., [16] generated a recombinant Shiga toxin 1-converting phage H-19B to facilitate the study of intestinal transmission of *stx*_1_-phages.

In the present work we have generated different recombinant phages with two antibiotic resistance genes to use them for different purposes. The use of these phages will allow analyse transduction in different matrices, as food or water samples. It would also be interesting to evaluate the phage induction and the transduction after different processes applied for food or water treatments, such as high temperature or high hydrostatic pressure (HHP), which has been reported as a method that can generate an increase in the induction of the lytic cycle of certain *stx*-phages [35].

The recombinant phages would be also useful tools to evaluate the ability of *stx*-phages to generate double lysogens and to evaluate whether the double lysogeny is really favoured in STEC, as some observations done in water environments or in strains isolated from humans and animals would suggest [15,33,36,37].

## Conclusion

The Red recombinase system was utilised and adapted for the purpose of disrupting chromosomal genes in *E. coli *K-12. Although it is a very simple and efficient system, once used in a chromosomal environment where some other phage-related sequences are present, several unexpected events were observed making it difficult to obtain recombinants. Additionally, the spontaneous release and loss of *stx *genes occurring in some strains carrying stx-prophages, as already described [38] decreases this method's efficacy.

Remarkably, this problem does not affect all *stx*_*2*_-encoding prophages, since some of them could be manipulated using the original protocol of Datsenko and Wanner [20]. Indeed, the substitution of the *stx2 *genes of φ330, a phage isolated from a Belgian *E. coli *O157:H7 isolate, by the chloramphenicol resistance cassette amplified from pKD3 was achieved in this way (S. Acosta, N. Buys and J.P. Hernalsteens, unpublished results).

The modification of the Red Recombinase system reported in the present study offer some solutions for constructing recombinant bacteriophages. This study focused on the construction of recombinant *stx*-phages carrying antibiotic resistance genes within the *stx *operon. However, the approach could also be considered to produce recombinant phages carrying some other marker genes such as: the β-galactosidase gene,*lacZ*; the bacterial luciferase gene, *luxAB*; or the green fluorescent protein gene,*gfp *[8]. In addition, this methodology could be useful for prophages infecting other enterobacteria, since the method has been applied to *Salmonella *[21]; *Shigella *[39]; *Serratia *[40]; and *Yersinia *[41], which also carry prophages encoding virulence genes [42-44].

## Methods

### Bacteriophages, bacterial strains, plasmids, and growth conditions

The bacteriophages ØA9, ØA312, ØA534, ØA549, ØA557, ØVTB55 used in the experiments were induced from selected STEC strains described elsewhere [36] and consisting of serotypes O157:H7 and O2:H27.

*E. coli *laboratory strains DH5α and C600 were used as host strains in some of the experiments described below. The aforementioned bacteriophages were introduced in these receptor strains as mentioned elsewhere [36] to generate the lysogens: C600(A9), DH5α(534), DH5α(557), DH5α(VTB55), DH5α(312) and DH5α(549).

Bacteriophage 933W [26] was induced from *E. coli *C600(933W), as described below, and used as a reference *stx*-bacteriophage.

Bacterial strains were grown in Luria-Bertani (LB) broth and on LB agar. The LB medium was supplemented with: ampicillin (Ap) (100 μg/ml); chloramphenicol (Cm) (5 μg/ml); and tetracycline (Tc)(5 μg/ml), when needed. SOB and SOC media [45] were used to prepare electrocompetent cells and for recovery after transformation. TSB medium supplemented with 5 mM CaCl_*2 *_was used for the preparation of phage lysates

### Plasmids

Plasmid pKD46 (Genbank AY048746) was used for the expression of Red recombinase [20]. Plasmid pACYC184 (Genbank X06403) [46] was used to obtain the tetracycline resistance gene (*tet*). Plasmid pKD3 (Genbank AY048742) [20] was used to obtain the chloramphenicol acetyl transferase gene (*cat*), which confers resistance to chloramphenicol. Plasmid pBC-SK+ (Stratagene Inc. Amsterdam, the Netherlands) was used as a control for the transformation. All vectors were purified using the Qiagen Plasmid Midi purification kit (Qiagen Inc., Valencia, USA).

### PCR techniques

PCR reactions were performed using a GeneAmp PCR system 2400 (Perkin-Elmer, PE Applied Biosystems, Barcelona, Spain.). One μl of cellular suspension obtained from three-four colonies was used for the PCR amplification. The oligonucleotides used in this study are described in Table 3.

Construction of the amplimers containing *tet *and *cat *genes, inserted in the *stx*_*2 *_gene, was performed as follows. The primer pairs Tc5'-Tc3'and Cm5-Cm3 (Table 3) were used for the amplification of the *tet *and *cat *genes respectively, with an annealing temperature of 54°C and elongation time of 45 s. Primer pairs amplified from the *stxA*_*2 *_subunit initial codon to the 5' region of each resistance cassette (5' fragment) and from the 3' region of each resistance cassette to the final codon of the *stxB*_*2 *_subunit (3' fragment) (Fig 1). The conditions used for all primer combinations were an annealing temperature of 41°C and elongation time of 45 s. The amplimers obtained were S2Aup/Tc5-stx (290 bp), S2Aup/Cm5-stx (291 bp) and GK4/Tc3-stx (281 bp) and GK4/Cm3-stx (281 bp).

Amplimers of the 5' fragment, the respective resistance gene and the 3' fragment for each resistance gene were annealed at their overlapping region (underlined letters in Table 3). They were then amplified by PCR as a single fragment with the external primers S2Aup and GK4 with an annealing temperature of 41°C and an elongation time of 2 min. The fusion product was amplified again using primer pair S2Aup/GK4. It was then excised from the gel and purified using the Qiaquick Gel Extraction Kit (Qiagen Inc., Valencia, USA). The final product was used for the transformation in the lysogens.

### Preparation of digoxigenin-labelled *stxA*_*2*_, *tc *and *cat *specific gene probes

A DNA fragment of the *stx*_*2*_*A *gene obtained with primers UP378/LP378, the *tet *gene and the *cat *gene, resulting from amplification with the respective primers (Table 3), were labeled with digoxigenin and used as probes. The probes were labeled by incorporating digoxigenin-11-deoxy-uridine-triphosphate (Roche Diagnostics, Barcelona, Spain) during PCR, as described in Muniesa *et al*., [36].

### Hybridization techniques

Plaque blot was performed using Nylon-N+ membranes (Hybond N+, Amersham Pharmacia Biotech, Spain) [47].

Colony hybridization was performed as previously described [37]. The membranes were washed in a solution consisting of 1% SDS, 2× SSC for five min at 50°C. They were then washed in 0.1% SDS, 2× SSC for 5 min at room temperature and finally in 2× SSC for 5 min at room temperature.

Membranes were pre-hybridized with standard pre-hybridization solution at 68°C for 2 hours. Stringent hybridization was achieved with the DIG DNA Labelling and Detection Kit (Roche Diagnostics, Barcelona, Spain), according to the manufacturer's instructions. The DIG-labeled probes were prepared as described above.

### Electroporation

Electroporation-competent cells were prepared from 10–50 ml of cultures in SOB medium and concentrated by centrifugation at 3000 × *g *for 5 min. They were then washed in 2 ml of ice-cold double distilled water. After four washing steps, the cells were suspended in 15–100 μl of ice-cold double-distilled water. The cells were mixed with the corresponding amount of DNA (plasmid or PCR-amplified, see results) in an ice-cold microcentrifuge tube and transferred to a 0.2 cm electroporation cuvette (Bio-Rad, Inc.). The cells were electroporated at 2.5 kV with 25 F and 200 ohm resistance. Immediately after electroporation, 1 ml of SOC medium [47] was added to the cuvette. The cells were transferred to a 17 by 100 mm polypropylene tube and recovered in SOC medium for 1–4 hours at either 30°C (for temperature-sensitive plasmids) or 37°C, without shaking. Cells were concentrated ten-fold from a 1 ml culture before plating on selective media.

### Recombinant phage construction

A protocol modified from the one-step inactivation method using the Red recombinase system, as proposed by Datsenko and Wanner [20], was performed to obtain recombinant phages. Antibiotic resistance cassettes were inserted inside the truncated *stx*_*2 *_gene of each phage to obtain recombinant phages carrying the resistance markers. For this purpose, plasmid pKD46 encoding the Red recombinase was transformed by electroporation, as described above. This was performed in electrocompetent cells prepared from 5 ml of the culture (approximately 5 × 10^9 ^CFU/ml) of lysogens: C600(A9), DH5α(534), DH5α(557), DH5α(VTB55), DH5α(312), DH5α(549) and C600(933W). Transformation of the vector was confirmed by PCR, using the primer pair RR46up/lp. In a second step, the transformation of 30 μl of each PCR amplimer (corresponding to 0.1–0.5 μg of amplified DNA) containing the *stx*_*2 *_gene, truncated by insertion of the respective resistance gene and prepared as described above (see PCR techniques), was performed in electrocompetent cells. These cells were prepared from 50 ml cultures (approximately 5 × 10^10 ^CFU/ml) of each lysogen containing the pKD46 plasmid, grown at 30°C in SOB medium with ampicillin and 0.1 M of L-arabinose to an OD_600 _of 0.6. After recovery in SOC medium and incubation for 4 hours, recombinant clones were selected on medium containing the appropriate antibiotic. Presumptive colonies were confirmed by PCR, using the *rho *primer and the respective primer for each antibiotic cassette. Cycling times and temperatures were according to the properties of the primer pairs. Positive clones were also further confirmed by sequencing.

### Sequencing of the *stx*_*2 *_gene, *cat *gene and *tet *gene encoded by temperate phages

PCR amplimers of the *stx *gene containing each antibiotic resistance gene and PCR amplimers obtained to characterise recombinant phages were sequenced. The oligonucleotides used for sequencing are described in Table 3.

Sequencing was performed with the ABI PRISM Big Dye III v.1 Terminator cycle Sequencing Ready reaction Kit (Perkin Elmer, Applied Biosystems, Spain) in an ABI PRISM 3700 DNA Analyzer (Perkin Elmer, Applied Biosystems, Spain), according to the manufacturer's instructions. All sequencing was performed in duplicate.

Nucleotide sequence analysis and searches for homologous DNA sequences in the EMBL and Genbank databases were performed with the GCG Wisconsin Package Version 10.2, Genetics Computer Group, Madison, Wisc. BLAST analyses were carried out with the tools available on the web [48].

### Isolation of temperate bacteriophages and preparation of phage lysates

Lysogens were grown from single colonies in TSB medium supplemented with 5 mM CaCl_2 _at 37°C to the exponential growth phase. Growth was measured with a spectrophotometer (Spectronic 501, Milton Roy. Belgium). At OD_600 _= 0.5, Mitomycin C was added to the cultures to a final concentration of 0.5 μg/ml. Cultures were then further incubated overnight. The induced cultures were centrifuged at 10,000 × *g *for 10 min and the supernatants were filtered through low protein-binding 0.22-μm-pore-size membrane filters (Millex-GP, Millipore, Bedford, MA).

### Bacteriophage transduction

To evaluate the transduction capacity of the recombinant phages, they were used to convert *E. coli *DH5α and/or C600 as previously described [9]. Antibiotic resistant colonies were tested by colony blot and confirmed by PCR amplification of the truncated *stx*_*2 *_gene containing the antibiotic marker.

## Abbreviations

Ap, ampicillin; *cat*, chloramphenicol acetyltransferase; Cm, chloramphenicol; *tet*, tetracycline resistance gene; Tc, tetracycline;

## Authors' contributions

SA performed experiments of hybridization. J-PH introduced the Red system method, supplied the plasmids and the strains. JJ supplied the host strains, generated Fig 1 and 2 and provided supervision. RSM performed the rest of the experiments. MM designed the oligonucleotides provided supervision and wrote the paper.

## Figures and Tables

**Table 1:** Transformation efficiency of the *E. coli *lysogens carrying *stx*_2_-phages

	pBC-SK+		pKD46	
	Vector +	*stx+*	Vector +	*stx+*
**DH5α (VTB55)**	99.0^a^	100.0	100	6
**DH5α (312)**	98.5	96.5	100	1
**DH5α (534)**	100.0	99.5	100	80
**DH5α (549)**	97.5	100.0	100	5
**DH5α (557)**	100.0	100.0	100	3
**C600(A9)**	99.2	100.0	100	100
**C600(933W)**	100.0	100.0	100	100

^a^Percentage of colonies positive for the presence of each vector performed in parallel in two plates, relative to the total number of colonies grown in plates with the respective antibiotic. Results were obtained by colony hybridization of the colonies present in a plate.

**Table 2:** Summary of the diverse conditions used and final protocol

**Step**^**a**^	**Condition**	**Obtention of recombinant colonies (CFU/ml)^**b**^**	**Final established condition**
Construction of the amplimer	Short homologous arms	No	Long primers
	Long homologous arms	Yes	Single PCR reaction for *tet*
	Single PCR reaction	21 for Tc	Three PCR reaction for *cat*
	Three PCR reactions	19 for Cm	
			
Amount of amplified DNA to be transformed	0.1 μg	0	0.5 μg
	0.25 μg	0	
	0.5 μg	21 Tc, 19 Cm	
			
Culture volume used for preparation of electrocompetent cells	5 ml (2 × 10^9 ^CFU/ml)	0	50 ml (5.10^10 ^CFU/ml)
	10 ml (10^10 ^CFU/ml)	0	
	25 ml (2.5 × 10^10 ^CFU/ml)	1 Tc, 0 Cm	
	50 ml (5 × 10^10 ^CFU/ml)	9 Tc, 8 Cm	
			
Temperature after electroporation	30°C	2 Tc, 1 Cm	37°C
	37°C	15 Tc, 9 Cm	
			
Arabinose concentration media	1 mM	18 Tc, 9 Cm	0.1 M
	10 mM	10 Tc, 8 Cm	
	0.1 M	20 Tc, 11 Cm	
			
Concentration of antibiotic in plating media	Tc 20 μg/ml, Cm 20 μg/ml	1 Tc, 0 Cm	Tc 5 μg/ml, Cm 5 μg/ml
	Tc 5 μg/ml, Cm 5 μg/ml	21 Tc, 19 Cm	

^a ^Each set of experiments was performed independently of the other tests with one phage for each antibiotic.

^b ^Phage used for *tet *was ØVTB55; Phage used for *cat *was ØA9.

**Table 3:** Oligonucleotides used in this study

**Primers**	**Sequence**	**Gene region**	**Size (bp)**	**Reference**
UP 378	GCGTTTTGACCATCTTCGT			
LP 378	ACAGGAGCAGTTTCAGACAG	378 bp fragment *stx*_*2*_-*A*	378	49
S2Aup	ATGAAGTGTATATTATTTA	Binds *stx2-A *subunit at initial codon	-	7
GK4	TCAGTCATTATTAAACTG	Binds *stx2-B *subunit at final codon	-	50
Cm 5	TGTGTAGGCTGGAGCTGCTTC			
Cm-3	CATATGAATATCCTCCTTAG	*cat *gene	1015	This study
Cm 5-stx	GAAGCAGCTCCAGCCTACACAACGAAGATGGTCAAAACGCG			
Cm3-stx	CTAAGGAGGATATTCATATGAGGAGTTAAGTATGAAGAAG	Used for construction of the 5' fragment and 3' fragment of the *cat *gene	-	This study
Tc 5	TCAGCCCCATACGATATAAG			
Tc-3	TGGAGTGGTGAATCCGTTAG	*tet *gene	1180	This study
Tc 5-stx	CTTATATCGTATGGGGCTGAACGAAGATGGTCAAAACGCG			
Tc3-stx	CTAACGGATTCACCACTCCAAGGAGTTAAGTATGAAGAAG	Used for construction of the 5' fragment and 3' fragment of the *tet *gene	-	This study
Tc-int	TGTCGGAATGGACGATAT	Bind Tc cassette at 100 bp of 5' of *tet*	-	This study
Rho	ATATCTGCGCCGGGTCTG	Binds *rho *independent terminator sequence upstream *stx*_*2*_-*A *subunit	-	This study
RR46 LP	GAGCTCTAAGGAGGTTAT			
RR46-UP	GTGCAGTACTCATTCGTT	Red recombinase gene in pKD46 vector	457	This study
pBC KS	TCGAGGTCGACGGTATC			
M13 rev	GGAAACAGCTATGACCATG	Detection of pBC-SK+ vector	158	Stratagene
